# Supplementary figures and images for: First insight into genetic diversity of two sympatric marten species between the Alps and Adriatic islands
Source: PLoS One. 2026 Apr 21;21(4):e0329925. doi: 10.1371/journal.pone.0329925 (PMC13098900; doi:10.1371/journal.pone.0329925)

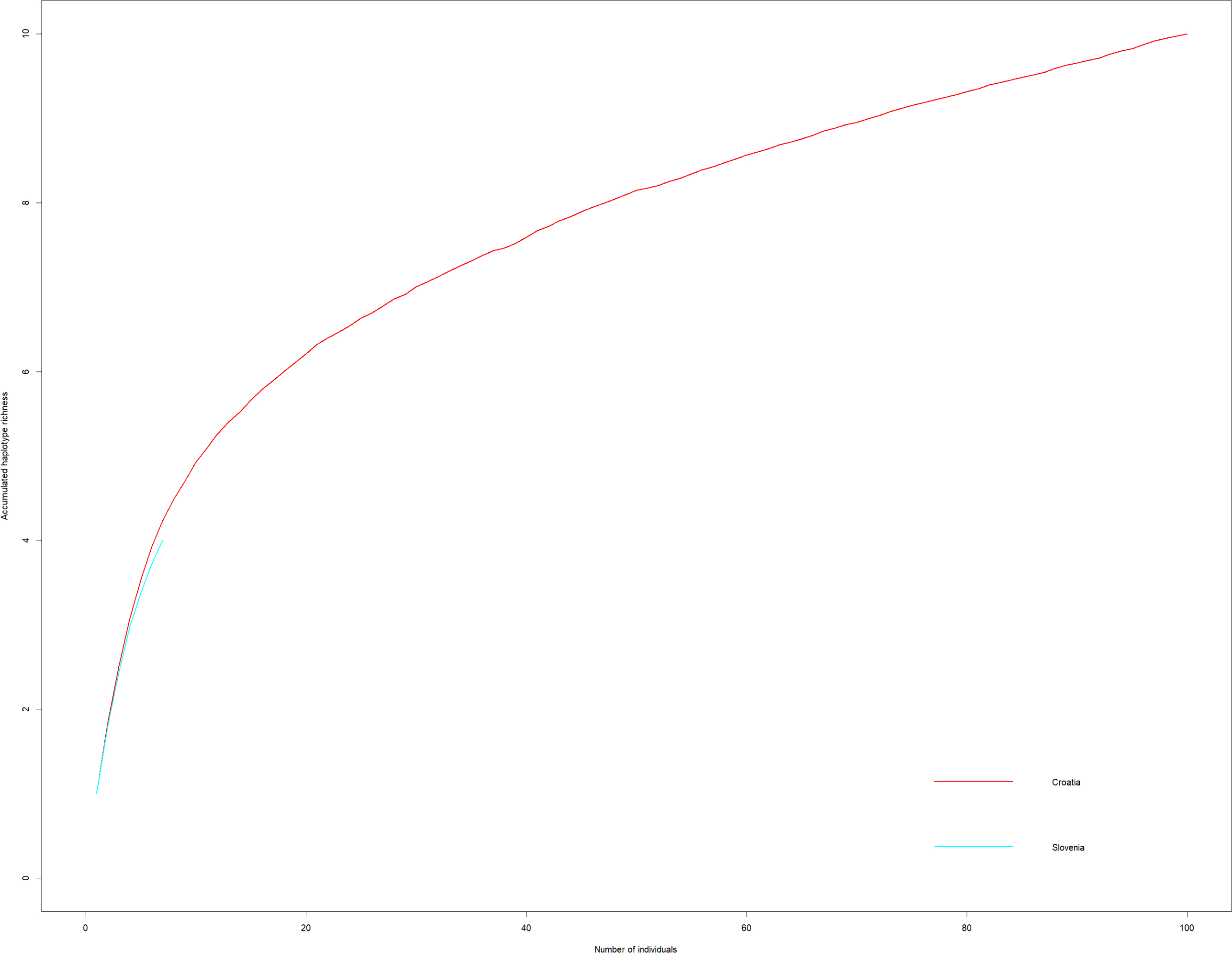

Supplement: S1 Fig — Haplotype accumulation curves by population of stone marten (Martes foina) samples from the present study from Croatia (red line) and Slovenia (cyan line). (TIF) [file pone.0329925.s001.tif]

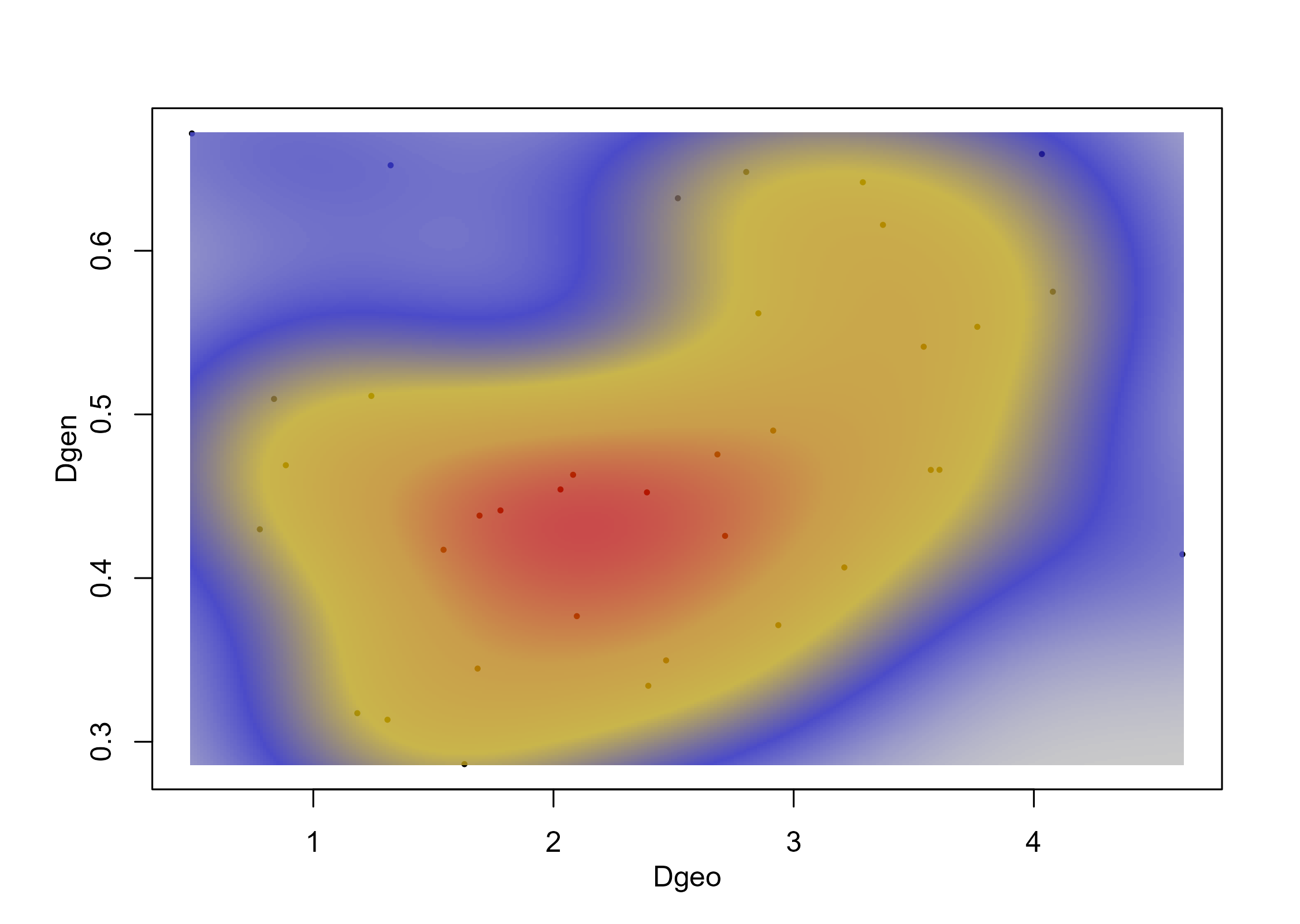

Supplement: S2 Fig — Relationship between genetic and geographic distances in Martes foina based on microsatellite data. The colour gradient represents the local density of data points (from light yellow = low density to dark blue = high density) obtained by two-dimensional kernel density estimation. No significant isolation by distance (IBD) was detected (Mantel test, p = 0.133); therefore, no regression line is shown. Dgeo – geographical distance (degrees); Dgen – genetic distance. (TIFF) [file pone.0329925.s002.tiff]
