# Supplementary material for: First insight into genetic diversity of two sympatric marten species between the Alps and Adriatic islands
Source: PLoS One. 2026 Apr 21;21(4):e0329925. doi: 10.1371/journal.pone.0329925 (PMC13098900; doi:10.1371/journal.pone.0329925)
Supplement: S1 Table — Basic data on pine marten (Martes martes) and stone marten (Martes foina) analysed samples included in the study. The “Haplotype” column represents the recognized haplotype, the “Haplotype – network” column represents how the haplotypes are labelled in the haplotype networks (Figs 2 and 6). (DOCX) [file pone.0329925.s003.docx]

**S1 Table. Sample data.** Basic data on pine marten (*Martes martes*) and stone marten (*Martes foina*) analysed in the study. The “Haplotype” column represents the recognized haplotype, the “Haplotype – network” column represents how the haplotypes are labelled in the haplotype networks (Fig 2 and Fig 6).

| **Sample ID** | **Species** | **Country** | **Latitude** | **Longitude** | **Haplotype** | **Haplotype - network** | **Microsatellite analysis** |
| --- | --- | --- | --- | --- | --- | --- | --- |
| LME1791 | *Martes foina* | Slovenia | 46.334 | 15.012 | MF4 | MF_Hap9 | Yes |
| LME1833 | *Martes foina* | Slovenia | 46.377 | 15.112 | MF2 | MF-1 | Yes |
| LME1834 | *Martes foina* | Slovenia | 46.365 | 15.058 | MF7 | MF_H26 | Yes |
| LME1835 | *Martes foina* | Slovenia | 45.956 | 14.418 | MF7 | MF_H26 | Yes |
| LME2322 | *Martes foina* | Slovenia | 46.339 | 14.995 | MF7 | MF_H26 | Yes |
| LME2323 | *Martes foina* | Slovenia | 46.368 | 15.080 | MF4 | MF_Hap9 | Yes |
| LME2614 | *Martes foina* | Slovenia | 45.537 | 13.865 |  |  | Yes |
| LME2616 | *Martes foina* | Slovenia | 45.519 | 13.747 | MF5 | MF_H15 | Yes |
| LME3223 | *Martes foina* | Croatia | 45.821 | 15.975 | MF5 | MF_H15 | Yes |
| LME3225 | *Martes foina* | Croatia | 45.181 | 14.724 |  |  | Yes |
| LME3226 | *Martes foina* | Croatia | 44.055 | 16.210 | MF7 | MF_H26 | Yes |
| LME3227 | *Martes foina* | Croatia | 45.262 | 13.744 | MF_H26 | MF_H26 | Yes |
| LME3228 | *Martes foina* | Croatia | 45.331 | 13.760 | MF4 | MF_Hap9 | Yes |
| LME3229 | *Martes foina* | Croatia | 45.226 | 14.014 |  |  | Yes |
| LME3230 | *Martes foina* | Croatia | 45.289 | 13.696 | MF4 | MF_Hap9 | Yes |
| LME3231 | *Martes foina* | Croatia | 45.259 | 13.609 |  |  | Yes |
| LME3232 | *Martes foina* | Croatia | 42.746 | 16.901 |  |  | Yes |
| LME3234 | *Martes foina* | Croatia | 45.085 | 19.066 | MF2 | MF-1 | Yes |
| LME3235 | *Martes foina* | Croatia | 46.026 | 17.278 |  |  | Yes |
| LME3236 | *Martes foina* | Croatia | 44.889 | 14.404 |  |  | Yes |
| LME3237 | *Martes foina* | Croatia | 45.821 | 15.975 | MF5 | MF_H15 | Yes |
| LME3238 | *Martes foina* | Croatia | 43.818 | 16.103 |  |  | Yes |
| LME3239 | *Martes foina* | Croatia | 45.259 | 17.826 | MF2 | MF-1 | Yes |
| LME3240 | *Martes foina* | Croatia | 44.398 | 15.688 | MF7 | MF_H26 | Yes |
| LME3241 | *Martes foina* | Croatia | 45.074 | 15.705 |  |  | Yes |
| LME3242 | *Martes foina* | Croatia | 45.378 | 18.332 | MF7 | MF_H26 | Yes |
| LME3243 | *Martes foina* | Croatia | 45.164 | 17.776 |  |  | Yes |
| LME3245 | *Martes foina* | Croatia | 45.821 | 15.975 | MF7 | MF_H26 | Yes |
| LME3383 | *Martes foina* | Croatia | 46.307 | 16.318 | MF2 | MF-1 | Yes |
| LME3384 | *Martes foina* | Croatia | 46.307 | 16.318 | MF2 | MF-1 | Yes |
| LME3385 | *Martes foina* | Croatia | 46.218 | 16.118 |  |  | Yes |
| LME3386 | *Martes foina* | Croatia | 46.307 | 16.318 | MF2 | MF-1 | Yes |
| LME3388 | *Martes foina* | Croatia | 46.218 | 16.118 | MF2 | MF-1 | Yes |
| LME3389 | *Martes foina* | Croatia | 46.218 | 16.118 |  |  | Yes |
| LME3390 | *Martes foina* | Croatia | 46.307 | 16.318 |  |  | Yes |
| LME3391 | *Martes foina* | Croatia | 46.218 | 16.118 |  |  | Yes |
| LME3394 | *Martes foina* | Croatia | 46.218 | 16.118 |  |  | Yes |
| LME3397 | *Martes foina* | Croatia | 45.821 | 15.975 | MF2 | MF-1 | Yes |
| LME3398 | *Martes foina* | Croatia | 45.821 | 15.975 |  |  | Yes |
| LME3400 | *Martes foina* | Croatia | 46.307 | 16.318 |  |  | Yes |
| LME3401 | *Martes foina* | Croatia | 46.307 | 16.318 |  |  | Yes |
| LME3402 | *Martes foina* | Croatia | 46.307 | 16.318 |  |  | Yes |
| LME3403 | *Martes foina* | Croatia | 46.307 | 16.318 |  |  | Yes |
| LME3404 | *Martes foina* | Croatia | 46.218 | 16.118 |  |  | Yes |
| LME3405 | *Martes foina* | Croatia | 46.218 | 16.118 |  |  | Yes |
| LME3406 | *Martes foina* | Croatia | 46.218 | 16.118 |  |  | Yes |
| LME3407 | *Martes foina* | Croatia | 46.218 | 16.118 | MF2 | MF-1 | Yes |
| LME3409 | *Martes foina* | Croatia | 46.307 | 16.318 |  |  | Yes |
| LME3413 | *Martes foina* | Croatia | 45.821 | 15.975 | MF7 | MF_H26 | Yes |
| LME3415 | *Martes foina* | Croatia | 45.485 | 16.361 | MF4 | MF_Hap9 | Yes |
| LME3416 | *Martes foina* | Croatia | 46.218 | 16.118 |  |  | Yes |
| LME3417 | *Martes foina* | Croatia | 46.218 | 16.118 |  |  | Yes |
| LME3418 | *Martes foina* | Croatia | 46.218 | 16.118 |  |  | Yes |
| LME3419 | *Martes foina* | Croatia | 46.307 | 16.318 |  |  | Yes |
| LME3420 | *Martes foina* | Croatia | 46.307 | 16.318 |  |  | Yes |
| LME3421 | *Martes foina* | Croatia | 46.307 | 16.318 | MF7 | MF_H26 | Yes |
| LME3424 | *Martes foina* | Croatia | 45.821 | 15.975 | MF2 | MF-1 | Yes |
| LME3425 | *Martes foina* | Croatia | 46.218 | 16.118 |  |  | Yes |
| LME3426 | *Martes foina* | Croatia | 46.218 | 16.118 |  |  | Yes |
| LME3430 | *Martes foina* | Croatia | 45.485 | 16.361 | MF1 | MF_Hap6 | Yes |
| LME3431 | *Martes foina* | Croatia | 45.821 | 15.975 | MF2 | MF-1 | Yes |
| LME3432 | *Martes foina* | Croatia | 46.307 | 16.318 | MF1 | MF_Hap6 | Yes |
| LME3433 | *Martes foina* | Croatia | 46.307 | 16.318 |  |  | Yes |
| LME3434 | *Martes foina* | Croatia | 45.821 | 15.975 |  |  | Yes |
| LME3435 | *Martes foina* | Croatia | 46.307 | 16.318 | MF2 | MF-1 | Yes |
| LME3436 | *Martes foina* | Croatia | 46.218 | 16.118 |  |  | Yes |
| LME3437 | *Martes foina* | Croatia | 46.307 | 16.318 |  |  | Yes |
| LME3438 | *Martes foina* | Croatia | 45.821 | 15.975 | MF2 | MF-1 | Yes |
| LME3439 | *Martes foina* | Croatia | 46.307 | 16.318 |  |  | Yes |
| LME3440 | *Martes foina* | Croatia | 46.026 | 15.896 | MF7 | MF_H26 | Yes |
| LME3441 | *Martes foina* | Croatia | 45.821 | 15.975 |  |  | Yes |
| LME3442 | *Martes foina* | Croatia | 46.218 | 16.118 | MF2 | MF-1 | Yes |
| LME3443 | *Martes foina* | Croatia | 46.218 | 16.118 |  |  | Yes |
| LME3444 | *Martes foina* | Croatia | 46.218 | 16.118 |  |  | Yes |
| LME3446 | *Martes foina* | Croatia | 46.307 | 16.318 |  |  | Yes |
| LME3447 | *Martes foina* | Croatia | 46.218 | 16.118 |  |  | Yes |
| LME3448 | *Martes foina* | Croatia | 46.218 | 16.118 |  |  | Yes |
| LME3449 | *Martes foina* | Croatia | 46.307 | 16.318 |  |  | Yes |
| LME3450 | *Martes foina* | Croatia | 46.307 | 16.318 |  |  | Yes |
| LME3451 | *Martes foina* | Croatia | 46.218 | 16.118 | MF2 | MF-1 | Yes |
| LME3452 | *Martes foina* | Croatia | 46.218 | 16.118 |  |  | Yes |
| LME3453 | *Martes foina* | Croatia | 46.307 | 16.318 |  |  | Yes |
| LME3455 | *Martes foina* | Croatia | 46.218 | 16.118 |  |  | Yes |
| LME3456 | *Martes foina* | Croatia | 46.307 | 16.318 |  |  | Yes |
| LME3459 | *Martes foina* | Croatia | 46.218 | 16.118 |  |  | Yes |
| LME3460 | *Martes foina* | Croatia | 46.307 | 16.318 | MF7 | MF_H26 | Yes |
| LME3462 | *Martes foina* | Croatia | 46.307 | 16.318 | MF2 | MF-1 | Yes |
| LME3463 | *Martes foina* | Croatia | 46.307 | 16.318 |  |  | Yes |
| LME3464 | *Martes foina* | Croatia | 46.307 | 16.318 | MF7 | MF_H26 | Yes |
| LME3465 | *Martes foina* | Croatia | 46.218 | 16.118 | MF1 | MF_Hap6 | Yes |
| LME3466 | *Martes foina* | Croatia | 45.821 | 15.975 | MF2 | MF-1 | Yes |
| LME3467 | *Martes foina* | Croatia | 45.821 | 15.975 | MF2 | MF-1 | Yes |
| LME3471 | *Martes foina* | Croatia | 46.218 | 16.118 | MF2 | MF-1 | Yes |
| LME3473 | *Martes foina* | Croatia | 43.160 | 16.604 |  |  | Yes |
| LME3474 | *Martes foina* | Croatia | 43.160 | 16.604 | MF4 | MF_Hap9 | Yes |
| LME3475 | *Martes foina* | Croatia | 43.160 | 16.604 |  |  | Yes |
| LME3476 | *Martes foina* | Croatia | 43.160 | 16.604 |  |  | Yes |
| LME3477 | *Martes foina* | Croatia | 43.160 | 16.604 |  |  | Yes |
| LME3478 | *Martes foina* | Croatia | 43.160 | 16.604 |  |  | Yes |
| LME3479 | *Martes foina* | Croatia | 43.160 | 16.604 |  |  | Yes |
| LME3480 | *Martes foina* | Croatia | 43.160 | 16.604 |  |  | Yes |
| LME3481 | *Martes foina* | Croatia | 43.160 | 16.604 |  |  | Yes |
| LME3482 | *Martes foina* | Croatia | 43.160 | 16.604 |  |  | Yes |
| LME3483 | *Martes foina* | Croatia | 43.160 | 16.604 | MF5 | MF_H15 | Yes |
| LME3484 | *Martes foina* | Croatia | 43.160 | 16.604 |  |  | Yes |
| LME3485 | *Martes foina* | Croatia | 43.160 | 16.604 |  |  | Yes |
| LME3486 | *Martes foina* | Croatia | 43.160 | 16.604 |  |  | Yes |
| LME3487 | *Martes foina* | Croatia | 43.160 | 16.604 |  |  | Yes |
| LME3488 | *Martes foina* | Croatia | 43.160 | 16.604 | MF4 | MF_Hap9 | Yes |
| LME3489 | *Martes foina* | Croatia | 43.160 | 16.604 |  |  | Yes |
| LME3490 | *Martes foina* | Croatia | 43.160 | 16.604 |  |  | Yes |
| LME3491 | *Martes foina* | Croatia | 43.160 | 16.604 | MF5 | MF_H15 | Yes |
| LME3493 | *Martes foina* | Croatia | 43.160 | 16.604 | MF4 | MF_Hap9 | Yes |
| LME3494 | *Martes foina* | Croatia | 43.160 | 16.604 |  |  | Yes |
| LME3495 | *Martes foina* | Croatia | 43.160 | 16.604 |  |  | Yes |
| LME3497 | *Martes foina* | Croatia | 43.160 | 16.604 | MF4 | MF_Hap9 | Yes |
| LME3499 | *Martes foina* | Croatia | 43.160 | 16.604 |  |  | Yes |
| LME3500 | *Martes foina* | Croatia | 43.160 | 16.604 |  |  | Yes |
| LME3501 | *Martes foina* | Croatia | 43.160 | 16.604 | MF4 | MF_Hap9 | Yes |
| LME3502 | *Martes foina* | Croatia | 43.160 | 16.604 | MF4 | MF_Hap9 | Yes |
| LME3503 | *Martes foina* | Croatia | 43.160 | 16.604 | MF4 | MF_Hap9 | Yes |
| LME3504 | *Martes foina* | Croatia | 43.160 | 16.604 | MF5 | MF_H15 | Yes |
| LME3505 | *Martes foina* | Croatia | 43.160 | 16.604 | MF4 | MF_Hap9 | Yes |
| LME3506 | *Martes foina* | Croatia | 43.160 | 16.604 | MF5 | MF_H15 | Yes |
| LME3507 | *Martes foina* | Croatia | 43.160 | 16.604 | MF5 | MF_H15 | Yes |
| LME3508 | *Martes foina* | Croatia | 43.160 | 16.604 | MF6 | MF6 | Yes |
| LME3509 | *Martes foina* | Croatia | 43.160 | 16.604 |  |  | Yes |
| LME3510 | *Martes foina* | Croatia | 43.160 | 16.604 | MF4 | MF_Hap9 | Yes |
| LME3511 | *Martes foina* | Croatia | 43.160 | 16.604 | MF4 | MF_Hap9 | Yes |
| LME3512 | *Martes foina* | Croatia | 43.160 | 16.604 | MF5 | MF_H15 | Yes |
| LME3513 | *Martes foina* | Croatia | 43.160 | 16.604 | MF4 | MF_Hap9 | Yes |
| LME3514 | *Martes foina* | Croatia | 43.160 | 16.604 | MF4 | MF_Hap9 | Yes |
| LME3517 | *Martes foina* | Croatia | 43.160 | 16.604 |  |  | Yes |
| LME3518 | *Martes foina* | Croatia | 43.160 | 16.604 |  |  | Yes |
| LME3519 | *Martes foina* | Croatia | 44.041 | 16.197 | MF7 | MF_H26 | Yes |
| LME3520 | *Martes foina* | Croatia | 46.146 | 15.671 | MF2 | MF-1 | Yes |
| LME3521 | *Martes foina* | Croatia | 46.146 | 15.671 | MF7 | MF_H26 | Yes |
| LME3522 | *Martes foina* | Croatia | 46.146 | 15.671 | MF7 | MF_H26 | Yes |
| LME3523 | *Martes foina* | Croatia | 46.146 | 15.671 | MF7 | MF_H26 | Yes |
| LME3524 | *Martes foina* | Croatia | 44.525 | 15.173 | MF7 | MF_H26 | Yes |
| LME3525 | *Martes foina* | Croatia | 44.525 | 15.173 | MF7 | MF_H26 | Yes |
| LME3526 | *Martes foina* | Croatia | 44.531 | 15.076 | MF7 | MF_H26 | Yes |
| LME3527 | *Martes foina* | Croatia | 44.531 | 15.076 | MF5 | MF_H15 | Yes |
| LME3528 | *Martes foina* | Croatia | 44.525 | 15.093 | MF7 | MF_H26 | Yes |
| LME3529 | *Martes foina* | Croatia | 43.373 | 16.416 | MF4 | MF_Hap9 | Yes |
| LME3530 | *Martes foina* | Croatia | 45.488 | 15.992 | MF1 | MF_Hap6 | Yes |
| LME3535 | *Martes foina* | Croatia | 45.156 | 17.994 | MF2 | MF-1 | Yes |
| LME3536 | *Martes foina* | Croatia | 45.156 | 17.994 | MF5 | MF_H15 | Yes |
| LME3537 | *Martes foina* | Croatia | 45.421 | 15.172 | MF8 | MF8 | Yes |
| LME3538 | *Martes foina* | Croatia | 45.323 | 13.569 | MF5 | MF_H15 | Yes |
| LME3539 | *Martes foina* | Croatia | 45.323 | 13.569 | MF5 | MF_H15 | Yes |
| LME3540 | *Martes foina* | Croatia | 43.981 | 16.360 | MF9 | MF9 | Yes |
| LME3541 | *Martes foina* | Croatia | 43.981 | 16.360 | MF9 | MF9 | Yes |
| LME3543 | *Martes foina* | Croatia | 43.703 | 16.638 | MF5 | MF_H15 | Yes |
| LME3544 | *Martes foina* | Croatia | 43.981 | 16.360 | MF9 | MF9 | Yes |
| LME3545 | *Martes foina* | Croatia | 46.218 | 16.118 | MF7 | MF_H26 | Yes |
| LME3546 | *Martes foina* | Croatia | 45.081 | 14.593 | MF7 | MF_H26 | Yes |
| LME3548 | *Martes foina* | Croatia | 45.371 | 15.074 | MF2 | MF-1 | Yes |
| LME3549 | *Martes foina* | Croatia | 45.605 | 15.721 | MF4 | MF_Hap9 | Yes |
| LME3550 | *Martes foina* | Croatia | 45.061 | 15.721 | MF7 | MF_H26 | Yes |
| LME3551 | *Martes foina* | Croatia | 45.089 | 14.587 | MF10 | MF10 | Yes |
| LME3552 | *Martes foina* | Croatia | 42.754 | 16.872 | MF5 | MF_H15 | Yes |
| LME3553 | *Martes foina* | Croatia | 45.371 | 15.074 | MF4 | MF_Hap9 | Yes |
| LME3554 | *Martes foina* | Croatia | 42.754 | 16.872 | MF5 | MF_H15 | Yes |
| LME3555 | *Martes foina* | Croatia | 44.984 | 15.066 | MF8 | MF8 | Yes |
| LME3557 | *Martes foina* | Croatia | 46.094 | 15.840 | MF2 | MF-1 | Yes |
| LME3558 | *Martes foina* | Croatia | 45.338 | 16.091 |  |  | Yes |
| LME3559 | *Martes foina* | Croatia | 45.672 | 15.651 |  |  | Yes |
| LME3560 | *Martes foina* | Croatia | 45.089 | 14.587 | MF5 | MF_H15 | Yes |
| LME3561 | *Martes foina* | Croatia | 44.874 | 14.982 | MF5 | MF_H15 | Yes |
| LME3562 | *Martes foina* | Croatia | 44.644 | 15.378 | MF7 | MF_H26 | Yes |
| LME3565 | *Martes foina* | Croatia | 45.285 | 14.605 | MF2 | MF-1 | Yes |
| LME3566 | *Martes foina* | Croatia | 45.672 | 15.651 | MF9 | MF9 | Yes |
| LME3568 | *Martes foina* | Croatia | 46.025 | 17.113 | MF2 | MF-1 | Yes |
| LME3570 | *Martes foina* | Croatia | 45.806 | 16.238 | MF2 | MF-1 | Yes |
| LME3571 | *Martes foina* | Croatia | 46.267 | 16.567 |  |  | Yes |
| LME3572 | *Martes foina* | Croatia | 45.821 | 15.975 |  |  | Yes |
| LME3573 | *Martes foina* | Croatia | 44.037 | 16.192 | MF9 | MF9 | Yes |
| LME3574 | *Martes foina* | Croatia | 43.909 | 16.400 | MF7 | MF_H26 | Yes |
| LME3575 | *Martes foina* | Croatia | 45.787 | 15.646 | MF4 | MF_Hap9 | Yes |
| LME3576 | *Martes foina* | Croatia | 46.208 | 16.044 |  |  | Yes |
| LME3577 | *Martes foina* | Croatia | 46.307 | 15.963 |  |  | Yes |
| LME3412 | *Martes martes* | Croatia | 45.821 | 15.975 | MM1 | Mm43 | Yes |
| LME3422 | *Martes martes* | Croatia | 46.218 | 16.118 | MM1 | Mm43 | Yes |
| LME3423 | *Martes martes* | Croatia | 46.218 | 16.118 | MM1 | Mm43 | Yes |
| LME3458 | *Martes martes* | Croatia | 46.307 | 16.318 | MM1 | Mm43 | Yes |
| LME3534 | *Martes martes* | Croatia | 45.054 | 19.030 | MM1 | Mm43 | Yes |
| LME3532 | *Martes martes* | Croatia | 45.571 | 18.697 | MM2 | MM2 | Yes |
| LME3395 | *Martes martes* | Croatia | 46.218 | 16.118 | MM3 | MM3 | Yes |
| LME3461 | *Martes martes* | Croatia | 46.307 | 16.318 | MM3 | MM3 | Yes |
| LME3531 | *Martes martes* | Croatia | 45.650 | 18.540 | MM3 | MM3 | Yes |
| LME3457 | *Martes martes* | Croatia | 45.485 | 16.361 | MM4 | Mm44 | Yes |
| LME3399 | *Martes martes* | Croatia | 46.307 | 16.318 | MM5 | MM5 | Yes |
| LME3469 | *Martes martes* | Croatia | 46.307 | 16.318 | MM5 | MM5 | Yes |
| LME3533 | *Martes martes* | Croatia | 45.440 | 17.762 | MM6 | MM6 | Yes |
| LME2615 | *Martes martes* | Slovenia | 46.321 | 15.003 | MM7 | Mm19 | Yes |
| LME3387 | *Martes martes* | Croatia | 46.307 | 16.318 | MM7 | Mm19 | Yes |
| LME3408 | *Martes martes* | Croatia | 46.218 | 16.118 | MM7 | Mm19 | Yes |
| LME3414 | *Martes martes* | Croatia | 45.485 | 16.361 | MM7 | Mm19 | Yes |
| LME3428 | *Martes martes* | Croatia | 46.307 | 16.318 | MM7 | Mm19 | Yes |
| LME3429 | *Martes martes* | Croatia | 46.307 | 16.318 | MM7 | Mm19 | Yes |
| LME3445 | *Martes martes* | Croatia | 46.218 | 16.118 | MM7 | Mm19 | Yes |
| LME3454 | *Martes martes* | Croatia | 45.821 | 15.975 | MM7 | Mm19 | Yes |
| LME3470 | *Martes martes* | Croatia | 46.218 | 16.118 | MM7 | Mm19 | Yes |
| LME3392 | *Martes martes* | Croatia | 46.218 | 16.118 | MM8 | Mm5 | Yes |
| LME3393 | *Martes martes* | Croatia | 46.218 | 16.118 | MM8 | Mm5 | Yes |
| LME3410 | *Martes martes* | Croatia | 46.307 | 16.318 | MM8 | Mm5 | Yes |
| LME3556 | *Martes martes* | Croatia | 45.897 | 16.856 | MM8 | Mm5 | Yes |
| LME3563 | *Martes martes* | Croatia | 45.740 | 16.617 | MM8 | Mm5 | Yes |
| LME3564 | *Martes martes* | Croatia | 45.897 | 16.856 | MM8 | Mm5 | Yes |
| LME3468 | *Martes martes* | Croatia | 46.307 | 16.318 |  |  | Yes |
