# Supplementary material for: First insight into genetic diversity of two sympatric marten species between the Alps and Adriatic islands
Source: PLoS One. 2026 Apr 21;21(4):e0329925. doi: 10.1371/journal.pone.0329925 (PMC13098900; doi:10.1371/journal.pone.0329925)
Supplement: S2 Table — The “Haplotype - GenBank” column represents the haplotype names obtained from GenBank, the “Haplotype – network” column represents how the haplotypes are labelled in the haplotype network (Fig 2). (DOCX) [file pone.0329925.s004.docx]

**S2 Table. *Martes foina* haplotypes obtained from GenBank**. The “Haplotype - GenBank” column represents the haplotype names obtained from GenBank, the “Haplotype – network” column represents how the haplotypes are labelled in the haplotype network (Fig 2).

| **Haplotype - GenBank** | **Haplotype - network** | **Region** | **GenBank ID** | **Reference** |
| --- | --- | --- | --- | --- |
| MF3 | MF_H26 | Croatia | KT734864 | Sindičić, 2015 |
| MF7 | MF_H26 | Croatia, Slovenia | KT734865 | Sindičić, 2015 |
| MF1 | MF_Hap6 | Croatia | KT734866 | Sindičić, 2015 |
| MF4 | MF_Hap9 | Croatia, Slovenia | KT734867 | Sindičić, 2015 |
| MF2 | MF-1 | Croatia, Slovenia | KT734869 | Sindičić, 2015 |
| MF5 | MF_H15 | Croatia, Slovenia | KT734870 | Sindičić, 2015 |
| MF8 | MF8 | Croatia | KT734871 | Sindičić, 2015 |
| MF10 | MF10 | Croatia | KT734872 | Sindičić, 2015 |
| MF6 | MF6 | Croatia | KT734873 | Sindičić, 2015 |
| MF9 | MF9 | Croatia | KT734874 | Sindičić, 2015 |
| HS1753 | MF_H26 | Germany | AB601564 | Hosoda et al. 2011 |
| MF01 | MF_H26 | Bulgaria | AB705456 | Nagai et al. 2012 |
| MF02 | MF02 | Bulgaria | AB705457 | Nagai et al. 2012 |
| MF03 | MF_Hap6 | Bulgaria | AB705458 | Nagai et al. 2012 |
| MF04 | MF04 | Bulgaria | AB705459 | Nagai et al. 2012 |
| MF_Hap1 | MF_H26 | Greece | KF898357 | Tsoupas et al. 2019 |
| MF_Hap2 | MF_H26 | Greece | KF898358 | Tsoupas et al. 2019 |
| MF_Hap3 | MF_H42 | Greece | KF898359 | Tsoupas et al. 2019 |
| MF_Hap4 | MF_Hap4 | Bulgaria | KF898360 | Tsoupas et al. 2019 |
| MF_Hap5 | MF_Hap5 | Greece | KF898361 | Tsoupas et al. 2019 |
| MF_Hap6 | MF_Hap6 | Greece | KF898362 | Tsoupas et al. 2019 |
| MF_Hap7 | MF_H37 | Greece | KF898363 | Tsoupas et al. 2019 |
| MF_Hap8 | MF_Hap6 | Greece | KF898364 | Tsoupas et al. 2019 |
| MF_Hap9 | MF_Hap9 | Greece | KF898365 | Tsoupas et al. 2019 |
| MF_Hap10 | MF_Hap10 | Greece | KF898366 | Tsoupas et al. 2019 |
| MF_Hap11 | MF_Hap11 | Greece | KF898367 | Tsoupas et al. 2019 |
| MF_Hap13 | MF_H33 | Greece | KF898368 | Tsoupas et al. 2019 |
| MF_Hap12 | MF_H33 | Greece | KF898369 | Tsoupas et al. 2019 |
| MF-1 | MF-1 | Ukraine, Russia | LC663748 | Ishii et al. 2023 |
| MF-2 | MF_H42 | Russia, Asia | LC663749 | Ishii et al. 2023 |
| MF-3 | MF-3 | Asia | LC663750 | Ishii et al. 2023 |
| MF-4 | MF-4 | Asia | LC663751 | Ishii et al. 2023 |
| MF-5 | MF-5 | Asia | LC663752 | Ishii et al. 2023 |
| MF-6 | MF-6 | Asia | LC663753 | Ishii et al. 2023 |
| MF-8 | MF-8 | Asia | LC663755 | Ishii et al. 2023 |
| MF-9 | MF-9 | Ukraine | LC663756 | Ishii et al. 2023 |
| MF-10 | MF-10 | Asia | LC663757 | Ishii et al. 2023 |
| MF-11 | MF-11 | Asia | LC663758 | Ishii et al. 2023 |
| MF-12 | MF-12 | Russia | LC663759 | Ishii et al. 2023 |
| MF-13 | MF_Hap9 | Russia | LC663760 | Ishii et al. 2023 |
| MF-15 | MF-15 | Russia | LC663761 | Ishii et al. 2023 |
| MF-16 | MF-16 | Asia | LC663762 | Ishii et al. 2023 |
| MF_H1 | MF_H1 | Eastern Turkey | MN651516 | Arslan et al. 2020 |
| MF_H2 | MF_H1 | Eastern Turkey | MN651517 | Arslan et al. 2020 |
| MF_H3 | MF_H1 | Eastern Turkey | MN651518 | Arslan et al. 2020 |
| MF_H4 | MF_H1 | Eastern Turkey | MN651519 | Arslan et al. 2020 |
| MF_H5 | MF_H1 | Eastern Turkey | MN651520 | Arslan et al. 2020 |
| MF_H6 | MF_H6 | Eastern Turkey | MN651521 | Arslan et al. 2020 |
| MF_H7 | MF_H7 | Eastern Turkey | MN651522 | Arslan et al. 2020 |
| MF_H8 | MF_H8 | Eastern Turkey | MN651523 | Arslan et al. 2020 |
| MF_H9 | MF_H1 | Eastern Turkey | MN651524 | Arslan et al. 2020 |
| MF_H10 | MF_H10 | Eastern Turkey | MN651525 | Arslan et al. 2020 |
| MF_H11 | MF_H10 | Eastern Turkey | MN651526 | Arslan et al. 2020 |
| MF_H12 | MF_H1 | Eastern Turkey | MN651527 | Arslan et al. 2020 |
| MF_H13 | MF_H1 | Eastern Turkey | MN651528 | Arslan et al. 2020 |
| MF_H14 | MF_H1 | Eastern Turkey | MN651529 | Arslan et al. 2020 |
| MF_H15 | MF_H15 | Eastern Turkey | MN651530 | Arslan et al. 2020 |
| MF_H16 | MF_H16 | Eastern Turkey | MN651531 | Arslan et al. 2020 |
| MF_H17 | MF_H16 | Eastern Turkey | MN651532 | Arslan et al. 2020 |
| MF_H18 | MF_H18 | Eastern Turkey | MN651533 | Arslan et al. 2020 |
| MF_H19 | MF_H19 | Eastern Turkey | MN651534 | Arslan et al. 2020 |
| MF_H20 | MF_H20 | Eastern Turkey | MN651535 | Arslan et al. 2020 |
| MF_H21 | MF_H20 | Eastern Turkey | MN651536 | Arslan et al. 2020 |
| MF_H22 | MF_H22 | Eastern Turkey | MN651537 | Arslan et al. 2020 |
| MF_H23 | MF_H23 | Eastern Turkey | MN651538 | Arslan et al. 2020 |
| MF_H24 | MF_H24 | Eastern Turkey | MN651539 | Arslan et al. 2020 |
| MF_H25 | MF_H1 | Eastern Turkey | MN651540 | Arslan et al. 2020 |
| MF_H26 | MF_H26 | Western Turkey | MN651541 | Arslan et al. 2020 |
| MF_H27 | MF_H26 | Western Turkey | MN651542 | Arslan et al. 2020 |
| MF_H28 | MF_H28 | Western Turkey | MN651543 | Arslan et al. 2020 |
| MF_H29 | MF_H29 | Western Turkey | MN651544 | Arslan et al. 2020 |
| MF_H30 | MF_H30 | Western Turkey | MN651545 | Arslan et al. 2020 |
| MF_H31 | MF_H26 | Western Turkey | MN651546 | Arslan et al. 2020 |
| MF_H32 | MF_H26 | Western Turkey | MN651547 | Arslan et al. 2020 |
| MF_H33 | MF_H33 | Western Turkey | MN651548 | Arslan et al. 2020 |
| MF_H34 | MF_H33 | Western Turkey | MN651549 | Arslan et al. 2020 |
| MF_H35 | MF_H35 | Western Turkey | MN651550 | Arslan et al. 2020 |
| MF_H36 | MF_H36 | Western Turkey | MN651551 | Arslan et al. 2020 |
| MF_H37 | MF_H37 | Western Turkey | MN651552 | Arslan et al. 2020 |
| MF_H38 | MF_H26 | Western Turkey | MN651553 | Arslan et al. 2020 |
| MF_H39 | MF_H26 | Western Turkey | MN651554 | Arslan et al. 2020 |
| MF_H40 | MF_H26 | Western Turkey | MN651555 | Arslan et al. 2020 |
| MF_H41 | MF_H26 | Western Turkey | MN651556 | Arslan et al. 2020 |
| MF_H42 | MF_H42 | Western Turkey | MN651557 | Arslan et al. 2020 |
| MF_H43 | MF_H43 | Western Turkey | MN651558 | Arslan et al. 2020 |
