# Supplementary material for: First insight into genetic diversity of two sympatric marten species between the Alps and Adriatic islands
Source: PLoS One. 2026 Apr 21;21(4):e0329925. doi: 10.1371/journal.pone.0329925 (PMC13098900; doi:10.1371/journal.pone.0329925)
Supplement: S3 Table — The “Haplotype - GenBank” column represents the haplotype names obtained from GenBank, the “Haplotype – network” column represents how the haplotypes are labelled in the haplotype network (Fig 2). (DOCX) [file pone.0329925.s005.docx]

**S3 Table. *Martes martes* haplotypes obtained from GenBank.** The “Haplotype - GenBank” column represents the haplotype names obtained from GenBank, the “Haplotype – network” column represents how the haplotypes are labelled in the haplotype network (Fig 2).

| **Haplotype - GenBank** | **Haplotype - network** | **Region** | **GenBank ID** | **Reference** |
| --- | --- | --- | --- | --- |
| MM1 | Mm1 | Balkan | KT734875 | Sindičić, 2015 |
| MM2 | MM2 | Balkan | KT734876 | Sindičić, 2015 |
| MM3 | MM3 | Balkan | KT734877 | Sindičić, 2015 |
| MM4 | MM4 | Balkan | KT734878 | Sindičić, 2015 |
| MM5 | Mm5 | Balkan | KT734879 | Sindičić, 2015 |
| MM6 | MM6 | Balkan | KT734880 | Sindičić, 2015 |
| MM7 | MM7 | Balkan | KT734881 | Sindičić, 2015 |
| MM8 | Mm8 | Balkan | KT734882 | Sindičić, 2015 |
| MM9 | MM9 | Balkan | KT734883 | Sindičić, 2015 |
| Mm1 | Mm1 | Iberia | HM025990 | Ruiz-González et al. 2013 |
| Mm2 | Mm2 | Iberia | HM025991 | Ruiz-González et al. 2013 |
| Mm3 | Mm3 | Iberia | HM025992 | Ruiz-González et al. 2013 |
| Mm4 | Mm4 | Iberia | HM025993 | Ruiz-González et al. 2013 |
| Mm5 | Mm5 | Iberia | HM025994 | Ruiz-González et al. 2013 |
| Mm6 | Mm6 | Iberia | HM025995 | Ruiz-González et al. 2013 |
| Mm7 | Mm7 | Iberia | HM025996 | Ruiz-González et al. 2013 |
| Mm8 | Mm8 | Iberia | HM025997 | Ruiz-González et al. 2013 |
| Mm9 | Mm9 | Central Europe, Iberia, Italy | HM025998 | Ruiz-González et al. 2013 |
| Mm10 | Mm10 | Italy | HM025999 | Ruiz-González et al. 2013 |
| Mm11 | Mm11 | Italy | HM026000 | Ruiz-González et al. 2013 |
| Mm12 | Mm12 | Italy | HM026001 | Ruiz-González et al. 2013 |
| Mm13 | Mm13 | Italy | HM026002 | Ruiz-González et al. 2013 |
| Mm14 | Mm14 | Italy | HM026003 | Ruiz-González et al. 2013 |
| Mm15 | Mm15 | Italy | HM026004 | Ruiz-González et al. 2013 |
| Mm16 | Mm16 | Italy | HM026005 | Ruiz-González et al. 2013 |
| Mm17 | Mm17 | Italy | HM026006 | Ruiz-González et al. 2013 |
| Mm18 | Mm18 | Central Europe, Italy, Scandinavia, Western Europe | HM026007 | Ruiz-González et al. 2013 |
| Mm19 | Mm19 | Balkan | HM026008 | Ruiz-González et al. 2013 |
| Mm20 | Mm20 | British Isles | HM026009 | Ruiz-González et al. 2013 |
| Mm21 | Mm21 | Western Europe | HM026010 | Ruiz-González et al. 2013 |
| Mm22 | Mm22 | Western Europe | HM026011 | Ruiz-González et al. 2013 |
| Mm23 | Mm23 | Baltics | HM026012 | Ruiz-González et al. 2013 |
| Mm24 | Mm24 | Central Europe | HM026013 | Ruiz-González et al. 2013 |
| Mm25 | Mm25 | Central Europe | HM026014 | Ruiz-González et al. 2013 |
| Mm26 | Mm26 | Western Europe | HM026015 | Ruiz-González et al. 2013 |
| Mm27 | Mm27 | Central Europe | HM026016 | Ruiz-González et al. 2013 |
| Mm28 | Mm28 | British Isles | HM026017 | Ruiz-González et al. 2013 |
| Mm29 | Mm29 | Central Europe, Western Europe | HM026018 | Ruiz-González et al. 2013 |
| Mm30 | Mm30 | Western Europe | HM026019 | Ruiz-González et al. 2013 |
| Mm31 | Mm31 | Central Europe, Scandinavia, Western Europe | HM026020 | Ruiz-González et al. 2013 |
| Mm32 | Mm32 | Central Europe | HM026021 | Ruiz-González et al. 2013 |
| Mm33 | Mm33 | Central Europe, Baltics | HM026022 | Ruiz-González et al. 2013 |
| Mm34 | Mm34 | Central Europe | HM026023 | Ruiz-González et al. 2013 |
| Mm35 | Mm35 | Central Europe | HM026024 | Ruiz-González et al. 2013 |
| Mm36 | Mm36 | Central Europe | HM026025 | Ruiz-González et al. 2013 |
| Mm37 | Mm37 | Central Europe | HM026026 | Ruiz-González et al. 2013 |
| Mm38 | Mm38 | Central Europe | HM026027 | Ruiz-González et al. 2013 |
| Mm39 | Mm39 | Balkan | HM026028 | Ruiz-González et al. 2013 |
| Mm40 | Mm40 | Baltics | HM026029 | Ruiz-González et al. 2013 |
| Mm41 | Mm41 | Central Europe | HM026030 | Ruiz-González et al. 2013 |
| Mm42 | Mm42 | Western Europe | HM026031 | Ruiz-González et al. 2013 |
| Mm43 | Mm43 | Central Europe | HM026032 | Ruiz-González et al. 2013 |
| Mm44 | Mm44 | Central Europe | HM026033 | Ruiz-González et al. 2013 |
| Mm45 | Mm45 | Scandinavia | HM026034 | Ruiz-González et al. 2013 |
| Mm46 | Mm46 | Scandinavia | HM026035 | Ruiz-González et al. 2013 |
| Mm47 | Mm47 | Italy | HM026036 | Ruiz-González et al. 2013 |
| Mm48 | Mm48 | Italy | HM026037 | Ruiz-González et al. 2013 |
| Mm49 | Mm49 | North-eastern Europe | HM026038 | Ruiz-González et al. 2013 |
| Mm50 | Mm50 | Baltics, North-eastern Europe | HM026039 | Ruiz-González et al. 2013 |
| Mm51 | Mm51 | North-eastern Europe | HM026040 | Ruiz-González et al. 2013 |
| Mm52 | Mm52 | North-eastern Europe | HM026041 | Ruiz-González et al. 2013 |
| Mm53 | Mm53 | North-eastern Europe | HM026042 | Ruiz-González et al. 2013 |
| Mm54 | Mm54 | North-eastern Europe | HM026043 | Ruiz-González et al. 2013 |
| Mm55 | Mm55 | North-eastern Europe | HM026044 | Ruiz-González et al. 2013 |
| Mm56 | Mm56 | North-eastern Europe | HM026045 | Ruiz-González et al. 2013 |
| Mm57 | Mm57 | North-eastern Europe | HM026046 | Ruiz-González et al. 2013 |
| Mm58 | Mm58 | North-eastern Europe | HM026047 | Ruiz-González et al. 2013 |
| Mm59 | Mm59 | North-eastern Europe | HM026048 | Ruiz-González et al. 2013 |
| Mm60 | Mm60 | North-eastern Europe | HM026049 | Ruiz-González et al. 2013 |
| Mm61 | Mm61 | North-eastern Europe | HM026050 | Ruiz-González et al. 2013 |
| Mm62 | Mm62 | North-eastern Europe | HM026051 | Ruiz-González et al. 2013 |
| Mm63 | Mm63 | North-eastern Europe | HM026052 | Ruiz-González et al. 2013 |
| Mm64 | Mm64 | North-eastern Europe | HM026053 | Ruiz-González et al. 2013 |
| Mm65 | Mm65 | North-eastern Europe | HM026054 | Ruiz-González et al. 2013 |
| Mm66 | Mm66 | North-eastern Europe | HM026055 | Ruiz-González et al. 2013 |
| Mm67 | Mm67 | Scandinavia | HM026056 | Ruiz-González et al. 2013 |
| Mm68 | Mm68 | Scandinavia | HM026057 | Ruiz-González et al. 2013 |
| Mm69 | Mm69 | Scandinavia | HM026058 | Ruiz-González et al. 2013 |
| 1422 | 1422 | Italy | OP374121 | Vecchioni et al. 2022 |
| 1449 | 1449 | Italy | OP374122 | Vecchioni et al. 2022 |
| 1466 | 1466 | Italy | OP374123 | Vecchioni et al. 2022 |
| 4124 | 4124 | Italy | OP374124 | Vecchioni et al. 2022 |
