# Supplementary material for: First insight into genetic diversity of two sympatric marten species between the Alps and Adriatic islands
Source: PLoS One. 2026 Apr 21;21(4):e0329925. doi: 10.1371/journal.pone.0329925 (PMC13098900; doi:10.1371/journal.pone.0329925)
Supplement: S4 Table — Microsatellite loci analysed in fragment analysis of pine marten (M. Martes) and stone marten (M. foina) published by Basto et al. 2010. (DOCX) [file pone.0329925.s006.docx]

**S4 Table. Microsatellite loci primers.** Microsatellite loci analyzed in fragmentation analysis of pine marten (*M. Martes*) and stone marten (*M. foina*) published by Basto et al. 2010.

| Primer pairs used | Sequence 5`-3` | Fluorescent labelling | Fragment length |
| --- | --- | --- | --- |
| Mf 1.11 | TTGCTGGCCCACATTGCA  CACAGGATATCCTGGAAC | NED | 219-223 bp |
| Mf 3.2 | TGTTAGCTTGCCCTATGC  GGACCCATGAAAAACAGT | FAM | 165–177 bp |
| Mf 2.13 | TTTTGCCAAGTCTTCTAG  GTAGAAAACAGGGACGGT | NED | 296-332 bp |
| Mf 6.5 | TCTTTTGGCTTTATCAGT  CTCACATGGGAAATAGTC | VIC | 249–265 bp |
| Mf 8.7 | AGTCACTATCTCATAGCT  GCATAGGACATTGGACTG | NED | 167–179 bp |
| Mf 4.17 | GAGGCGACAAATTCCGGT  CCAAGCTTCAGAATCTAG | FAM | 215–243 bp |
| Mf 1.18 | CTTGAATTAGCTGTGTCA  AGTCCCTATGATTGCATG | VIC | 158-174 bp |
| Mf 3.7 | AAGATTTTAACCACCATG  GCCTGTTATCAGCCAGCT | VIC | 165-221 bp |
| Mf 8.10 | ATCTGTATTATCTGCATA  CCAAAGTGTGATGTTTGC | NED | 151–167 bp |
| Mf 1.1 | CTGTGTCAGAAAATGTGC  AGGGCTGGTAATACCATG | FAM | 174–182 bp |
| Mf 4.10 | CCAGGGTCCTGGGACTG  GAGAACTGTGGTTTTCAC | NED | 332–352 bp |
| Mf 8.8 | GGAAGAGGTGATTTCTGA  TCAGCTGGCGATCAGAGT | PET | 230-274 bp |
| Mf 1.3 | TTGCTGGAGGTGACCTTG  CTGGGATTGAGCCTTGCA | NED | 221–237 bp |
