# Supplementary material for: First insight into genetic diversity of two sympatric marten species between the Alps and Adriatic islands
Source: PLoS One. 2026 Apr 21;21(4):e0329925. doi: 10.1371/journal.pone.0329925 (PMC13098900; doi:10.1371/journal.pone.0329925)
Supplement: S5 Table — (DOCX) [file pone.0329925.s007.docx]

**S5 Table.** Genetic variation in the analysed samples of stone marten.

|  | **P** | **h** | **Hd (SD)** | **π (SD)** | **Tajima’s D** |
| --- | --- | --- | --- | --- | --- |
| Overall (n=104) | 9 | 10 | 0.811 (0.015) | 0.005 (0.001) | 1.528 (p>0.10) |
| Slovenia (n=7) | 6 | 4 | 0.810 (0.039) | 0.005 (0.002) | -0.339 (p>0.10) |
| Croatia (n=97) | 9 | 10 | 0.814 (0.015) | 0.006 (0.001) | 1.540 (p>0.10) |

P: number of polymorphic sites, h: number of haplotypes, Hd: haplotype diversity, π: nucleotide diversity, SD: standard deviation.
