# Supplementary material for: First insight into genetic diversity of two sympatric marten species between the Alps and Adriatic islands
Source: PLoS One. 2026 Apr 21;21(4):e0329925. doi: 10.1371/journal.pone.0329925 (PMC13098900; doi:10.1371/journal.pone.0329925)
Supplement: Attachment — Submitted filename: Reviewer #2_05.11.2025.docx [file pone.0329925.s008.docx]

**Reviewer #2**

#1: In this manuscript, the authors use mtDNA and nuclear markers to investigate how two mesocarnivores, the pine marten (Martes martes) and the stone marten (Martes foina), differ in their genetic structure, diversity, and connectivity across Croatia and Slovenia. They assess genetic patterns of differentiation in ~190 stone marten and 28 pine marten individuals sampled from mainland and island populations. The results seem to reveal sub-structuring in the stone marten, mainly between island and mainland populations, while pine marten sub-structuring remains unclear. While I think this paper has a good starting dataset and a lot of potential, I have several large comments that would need to be addressed before submission, mainly regarding the choice of groupings for the methods as well as the methods themselves. Additionally, the manuscript currently reads very choppy and is confusing and lengthy as a reader. All of this is addressed below.

**Reply #1: We appreciate the reviewer’s feedback regarding the manuscript’s readability. We did our best to carefully revise the manuscript to improve its clarity, flow, and conciseness.**

#2 Abstract: the abstract claims “For pine marten, we found a significant genetic structuring, with pronounced differentiation between island and mainland populations, and a further substructure within the mainland. No significant isolation by distance was detected (Mantel test, p = 0.15), suggesting that genetic differentiation is driven more by habitat discontinuities and anthropogenic barriers rather than geographical distance alone. In contrast, stone marten exhibited weak genetic structure and high genetic diversity, indicating gene flow and potential landscape permeability for this more synanthropic species. These contrasting pattern underscore species-specific responses to landscape fragmentation and highlight the need to tailor management strategies accordingly.” But the structuring between island and mainland is in the stone marten for the results and discussion? Also in the discussion there is no follow-up on the pine marten distribution, etc. which is them confusing to add to the abstract. This abstract needs to be re-written to follow what is discussed in the manuscript.

**Reply #2: Thank you for this important comment. We have rewritten the Abstract to correctly attribute the island–mainland genetic structuring to stone marten, clarify that the pine marten results are based on a smaller mtDNA dataset with limited spatial resolution, and have removed unsupported references to anthropogenic barriers. The revised version now accurately reflects the analyses and interpretations presented in the Results and Discussion sections.**

#3: I think there is a lot of good information in the introduction, I just think it needs to be re-written substantially. I think a broader importance of looking at these two species should be highlighted first in the introduction before narrowing the focus. The authors mention that the stone marten is more ‘adaptable’ and the pine marten maybe more ‘sensitive’ to human change etc. I think this is a better set-up to the paper than it currently has. What can we learn about these two species that have different requirements. How do these different behaviors/requirements impact the degree of genetic differentiation across populations, etc. Would we expect to see differences? We would certainly expect to see differences sooner in the pine marten if there is substantial human impact. As I am not an expert in this species, I think the authors would benefit from considering how their study can inform wider marten research and connect their study to a broader viewpoint. Additionally, there is a lot of information on other studies, which is great, but as a reader it feels as if it’s a lot of information without a clear thread that leads to why this study in Croatia and Slovenia is necessary and how it contributes something novel. The authors should think to make it more streamlined, where a reader without a history in marten research can follow along.

**Reply #3: The Introduction has been revised to highlight the broader ecological importance of comparing the two Martes species, emphasizing their contrasting adaptability and expected genetic responses to human impact. We streamlined the background to focus on studies most relevant to our aims, removed detailed information on hunting and roadkills, and clarified the novelty of studying populations in Croatia and Slovenia.**

#4 Line 39: “spatial genetic distribution in sympatry”. I think this should be expressed differently since sympatry inherently implies overlapping spatial ranges and genetic distribution, I am not sure what is meant by this.

**Reply #4: Thank you for the suggestion. We have revised the sentence to replace “spatial genetic distribution in sympatry” with “genetic structure in areas of sympatry” for clarity.**

#5 Line 104-110: These few lines are a bit confusing. I would restate as:
Between 2014-2024, the annual harvest of stone marten ranged from 790 (2020) to 995 (2015) individuals, and pine marten between 82 (2024) and 127 (2017). During this same period, registered roadkill ranged from 342 and 24 (2022) to 430 and 49 (2019) for stone and pine marten, respectively

**Reply #5: We rewrote the Introduction according to the editor’s and Reviewer 1’s suggestions, so all this information has been excluded.**

#6 My main issue is that several of the analyses are they are based on arbitrary country assignments, which in the introduction, the authors state there are no clear barriers since there are large corridors of uninterrupted forested land.

**Reply #6: We conducted the analyses at the country level because management and conservation of both marten species are implemented nationally, and this scale provides results directly relevant for management decisions. Moreover, our sampling coverage was not dense or continuous enough to define smaller, cross-border population units with confidence. Considering the dispersal capacity of both species and the geographical distances between most sampling sites across Croatia and Slovenia, we treated these as separate population groups. Therefore, country-based grouping was the most practical and biologically reasonable approach for the available data.**

#7 Additionally, I think a PCA should be used instead of a DAPC, since this is not biased by sampling locations. A PCA analysis should also be carried out on the pine marten, even with low sample size, because a PCA makes no a priori assumptions on clusters, it is still an option to run this here. Clusters should be chosen after running PCA and defining clusters. Additionally, the authors should look at how these genetic clusters translate to spatial groups and report on this.

**Reply #7: We agree that PCA is more appropriate and have replaced the DAPC with PCA combined with data-driven clustering for stone marten. We did not perform PCA for pine marten, as only a few samples were included in the study, which prevents meaningful population genetic analysis based on msat data. The reviewer likely did not notice that microsatellite analyses were not conducted for pine marten.**

#8 General comment: the results are currently extremely long and un-structured. I would only keep what is necessary to convey the main points that are presented in the tables and figures as currently, it feels repetitive and long for what is actually being presented in the figures/tables.

**Reply #8: We followed more specific recommendations from Reviewer 1 and excluded the repetitive parts.**

#9 202-203: why did some samples fail?

**Reply #9: Most of the failed samples originated from road-killed individuals, where DNA degradation was extensive due to exposure to environmental conditions prior to collection. Consequently, the extracted DNA often contained PCR inhibitors and was of insufficient quality or quantity for successful amplification, which explains the failure of these samples in genetic analyses.**

#10: 206: It would be nice here to remind the reader of how many stone marten individuals you have per country.

**Reply #10: We have revised the sentence to include the number of samples from each country.**

#11: 216-222: here the authors talk about differences between Slovenia and Croatia, but previously in the introduction talk about how the habitat is very well connected in this region. Therefore, it seems to me that a better way to define populations is by known barriers and or performing a PCA on the genetic data to see which clusters appear.

**Reply #11: Our statement about a “well-connected” habitat refers to landscape (structural) connectivity, not proven genetic panmixia. With our non-dense, discontinuous sampling, data-driven structure (PCA) showed only an island vs. mainland split and no stable cross-border clusters. Because management is implemented nationally and our sampling cannot robustly define smaller units, we analyzed at the country level as the most practical and defensible choice for this dataset.**

#12: Line 220-222: These sentences are not understandable as a reader

**Reply #12: We have rewritten the sentences to improve clarity and readability.**

#13 Figure 1: I believe the map was cut off from my view, but in general, it would be good to add a background to the spatial map of forest cover as this was mentioned as being important for dispersal/movement through habitat.
**Reply #13: We added an elevation model, selected color‑blind‑safe palettes with high contrast and ensured legends/captions.**

#14 Figure 2: colors between certain groups are too similar to make clear distinctions between them

**Reply #14: We changed some colors to be more distinguishable.**

#15: Line 280-289 and Figure 3: There are numbered clusters in the figured which are described no where in the text or figure caption. I disagree with the use of the DAPC, and would suggest a normal PCA, but it does seem that there are at least 3 clear genetic clusters from their results. Which it is unclear if this falls along the Croatia/Slovenia sampling line since there is no description or explanation. However, I would suggest that the authors use these clusters to define groups to run different analyses on and also look at how the groups resulting from PCA are spatially structured. I also generally think the authors should take the time to make the figures outside of the programs running the analysis to have a better looking more concise figure. Figure 1,2 and 3 could be combined into one figure where the clusters from a PCA can be mapped onto the sampling map and then linked to the mtDNA and nuclear DNA. See Lucena-Perez et al. 2020 for an example. Spending the time to do this greatly enhances reader comprehension and translates better the important results. As it reads currently, I as a reader am trying to find the important points from the results.

**Reply #15: We replaced DAPC analysis with PCA.**

#16: Table 17. Finally, the clusters identified are used to look at FST, which does show clear differences. It is therefore clear that using the Croatia/Slovenia split is simply not informative and unnecessary.

**Reply #16: We agree that population definition should ideally reflect biological and landscape realities. However, even though PCA analyses show some differentiation between island and mainland populations, our management framework operates at the national level. Because the habitat is well connected across the Croatia–Slovenia border and our sampling is not dense enough to robustly define finer-scale genetic units, analyzing populations at the country level remains the most practical and management-relevant approach.**

#17 Line 321-324: seems more like discussion

**Reply #17: Moved to discussion.**

#18 Figure 5. Unnecessary

**Reply #18: We moved the figure into Supplementary material (S2 Fig).**

#19 Line 326-330: this does not need its own sub-section, this can be combined with the above results as a secondary test to FST etc.

**Reply #19: We revised the text as it was suggested.**

#20 Line 333: I think the results from stone and pine marten should be presented together, as the analysis is a similar framework and it makes the results extremely long to keep them separate. Again here, I suggest PCA results to be done first to define clusters and all other analyses be done based on those identified clusters.

**Reply #20: We respectfully disagree with combining the stone marten and pine marten analyses. Although the analytical framework is similar, the two species occupy different habitats and show distinct population structures. The pine marten is absent from the islands, whereas the stone marten occurs both on the mainland and islands, reflecting different ecological and phylogeographic patterns. For this reason, we consider it more appropriate and biologically meaningful to present the results separately, maintaining the phylogeographic pattern between the two species.**

#21: Line 393-402: my general question here, is how are these mtDNA haplotypes distributed in space. It is clear you have samples belonging to the Eurasian lineage and from eastern Turkey. Are they distributed differently in space? Are they mixed? This might give some insight into how these two lineages meet in this space.

**Reply #21: We have added a description of the spatial distribution of the mtDNA lineages. The revised text now clarifies that haplotypes belonging to the Major Eurasian group occur across both Croatia and Slovenia, whereas those clustering with the eastern Turkish lineage are restricted to southern Croatia, suggesting a possible secondary contact zone in the northern Balkans.**

#22: Line 407: “there is evidence for to the existence”

**Reply #22: We rephrased the text.**

#23: Line 424-431: I am confused how if the Tajimas D were not significant the mtDNA diversity can be enoguh for demographic growth scenarios. In what time frame? Since the LGM? This seems quite evident in most species in Europe, so then the question is not, did they expand, but from where did they expand? I would be a bit more clear on what exactly from the results you are using to support your claims, and also be more clear with what questions you want to and can answer from your data.

**Reply #23: We have clarified the interpretation of demographic expansion by emphasizing that it is primarily supported by the significantly negative Fu’s Fs values and the star-like haplotype network structure, while Tajima’s D was not significant. We also specified that these signals are consistent with post-LGM expansion from southern refugia in the Balkans and Anatolia, as discussed in previous phylogeographic studies.**

#24 Line 435-440: so if it is known already there are no barriers between countries, this calls into question why half of the analyses are carried out using the countries separately?
**Reply #24: We already explained in the comment above.**

#25 Line 449-454: This is a great summary of what is going on. I think the methods and results should follow this framework of trying to identify where there may be breaks in the gene flow between groups – and quantify how much.

**Reply #25: We tried to rephase other sections according to this comment.**

#26: Then pine martens are completely left out of the rest of the discussion. I think there may still be valuable insight for a first look into connectivity, specifically if a PCA is carried out. And it can be discussed, even with limitations, that the first look shows X, but more samples and follow-up would be needed to determine if there are distinct clusters or groups and if it is experiencing fragmentation as it is the more sensitive species..

**Reply #26: We did not perform a PCA for this species because we did not conduct a microsatellite analysis, as there were too few successful samples to allow a meaningful population-level analysis. Given these limitations, any inference about connectivity or population structure would be unreliable. We therefore chose not to include any possibilities for speculative interpretations and instead focused on the stone marten results, for which the data quality and sample size were sufficient for robust analysis.**
