# Supplementary material for: First insight into genetic diversity of two sympatric marten species between the Alps and Adriatic islands
Source: PLoS One. 2026 Apr 21;21(4):e0329925. doi: 10.1371/journal.pone.0329925 (PMC13098900; doi:10.1371/journal.pone.0329925)
Supplement: Attachment — Submitted filename: Reviewer#2_26.2.2026.docx [file pone.0329925.s009.docx]

**Reviewer #2:**

#1: I believe this manuscript is now ready for acceptance. While the previous version already rested on a solid foundation in terms of sampling, analyses, and overall study design, the revised manuscript represents a substantial improvement in clarity, structure, and accessibility. In particular, the revised introduction provides a much clearer and more engaging framework for the study, effectively setting up both the biological motivation and the broader relevance of the work. The research questions are now articulated in a way that is easy to follow, even for readers who may not be specialists in the specific system or methods used.

**We appreciate the positive assessment that the revised manuscript is now ready for acceptance. We are pleased that the revised Introduction provides a clearer and more engaging framework, and that the biological motivation, broader relevance, and research questions are now articulated in an accessible manner for both specialists and non-specialists.**

#2: The authors have done an excellent job of explaining why this study matters, both within the context of the focal system and for the wider field. By more clearly linking the results to overarching concepts and ongoing debates, the manuscript now communicates its significance beyond a narrow audience. This improved framing allows the reader to better appreciate how the analyses contribute to current understanding and why the findings are timely and relevant.

**We acknowledge the positive evaluation of the improved framing of the study’s significance within the focal system and the wider field. We are glad that the revised text more clearly links our findings to broader concepts and ongoing debates, and that this helps convey the timeliness and relevance of the work to a broader audience.**

#3: Overall, the revisions have transformed a technically strong but previously dense manuscript into a clear, coherent, and compelling piece of work. The study is now accessible to a broader scientific audience without sacrificing rigor or depth, which greatly strengthens its impact. For these reasons, I fully support acceptance of the manuscript in its current form.

**We appreciate the conclusion that the revisions have improved clarity, coherence, and accessibility without reducing rigor. We note the full support for acceptance of the manuscript in its current form.**
